# Predictors of Hospitalization in Veterans with COVID 19 Infection

**DOI:** 10.1093/geroni/igaa057.3525

**Published:** 2020-12-16

**Authors:** Jorge Ruiz, Sergio Ruiz, Marlena Fernandez, Victor Cevallos, Yaseen Jumani, Manasa Morisetti, Sahar Hasanain, Michael Mintzer

**Affiliations:** Miami VA Healthcare System, Miami, Florida, United States

## Abstract

Frailty, a clinical syndrome characterized by vulnerability to stressors resulting from a loss of physiological reserve across multiple systems, is a common condition that affects older adults. In patients with COVID 19 infection, frailty may place older adults at higher risk for poor clinical outcomes including hospitalizations. The aim of this study was to determine independent variables associated with all-cause hospitalization in Veterans with COVID 19 infection. This was a cross-sectional study nested within a retrospective cohort study of Veterans who contracted COVID 19 as detected by PCR. A VA Frailty Index (VA-FI) was generated at baseline as a proportion of morbidity, function, sensory loss, cognition and mood and other variables from electronic health records. The VA-FI categorized Veterans into non-frail (FI<.21) and frail (FI≥.21). At the time or after the COVID 19 testing, hospitalization data was aggregated, and proposed variables were subjected to stepwise backward logistic regression. Likelihood ratios were obtained, and those with p<0.05 were identified as independent risk factors for hospitalization. A total of 2621 Veterans with COVID 19 infection were identified during the study period with a total of 650 hospitalizations. Mean age was 54.99 (SD=16.71) years, 59.80% White, 87.60% male, 25.10% frail. The independent factors associated with hospitalization upon backward logistic regression were age (OR=1.049, 95%CI:1.041-1.057), frailty (OR=2.350, 95%CI: 1.797-3.074), African American race (OR=1.467, 95%CI:1.188-1.812), schizophrenia (OR=0.401, 95%CI:0.235-0.686), and substance abuse (OR=0.602, 95%CI:0.476-0.762). In veterans with COVID 19 infection, age, frailty, race (African American), schizophrenia, and substance abuse were predictors of all-cause hospitalization.

